# Nanoprecipitate‐Strengthened High‐Entropy Alloys

**DOI:** 10.1002/advs.202100870

**Published:** 2021-10-22

**Authors:** Liyuan Liu, Yang Zhang, Jihong Han, Xiyu Wang, Wenqing Jiang, Chain‐Tsuan Liu, Zhongwu Zhang, Peter K. Liaw

**Affiliations:** ^1^ Key Laboratory of Superlight Materials and Surface Technology Ministry of Education College of Materials Science and Chemical Engineering Harbin Engineering University Harbin 150001 China; ^2^ Department of Materials Science and Engineering College of Engineering City University of Hong Kong Hong Kong 999077 China; ^3^ Department of Materials Science and Engineering The University of Tennessee Knoxville TN 37996‐2100 USA

**Keywords:** alloy design, high‐entropy alloys, mechanical properties, nanoprecipitates, precipitation strengthening

## Abstract

Multicomponent high‐entropy alloys (HEAs) can be tuned to a simple phase with some unique alloy characteristics. HEAs with body‐centered‐cubic (BCC) or hexagonal‐close‐packed (HCP) structures are proven to possess high strength and hardness but low ductility. The faced‐centered‐cubic (FCC) HEAs present considerable ductility, excellent corrosion and radiation resistance. However, their strengths are relatively low. Therefore, the strategy of strengthening the ductile FCC matrix phase is usually adopted to design HEAs with excellent performance. Among various strengthening methods, precipitation strengthening plays a dazzling role since the characteristics of multiple principal elements and slow diffusion effect of elements in HEAs provide a chance to form fine and stable nanoscale precipitates, pushing the strengths of the alloys to new high levels. This paper summarizes and review the recent progress in nanoprecipitate‐strengthened HEAs and their strengthening mechanisms. The alloy‐design strategies and control of the nanoscale precipitates in HEAs are highlighted. The future works on the related aspects are outlined.

## Graphical abstract

This paper summarizes and reviews the recent progress in nanoprecipitate‐strengthened high‐entropy alloys (HEAs) and their strengthening mechanisms. The formation of precipitates in HEAs and their effects on the mechanical properties are addressed and discussed. The alloy‐design strategies and control of the nanoscale precipitates in HEAs are highlighted. The future works on the related aspects are outlined.

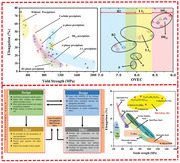

## 1 Introduction

Traditional alloys usually use one element as the main component, and property improvement is mainly based on adding a small amount of other alloying elements.^[^ ^1^, ^2^, ^3^, ^4^, ^5^, ^6^, ^7^, ^8^ ^]^ According to the Gibbs Phase Rule, the excessive addition of alloy elements will cause the formation of some intermetallic compounds in the alloys. Once the brittle compounds are formed, it will seriously affect the performance of the alloys, leading to the performance deterioration. Therefore, in the design concept of traditional alloys, when properties of alloys satisfy the application requirement, the types of alloy elements should be reduced as few as possible.^[^ ^9^, ^10^, ^11^ ^]^

However, the design concept of high‐entropy alloys (HEAs) is thoroughly different from the traditional alloys. Since Yeh and Cantor first proposed the concept of HEAs in 2004,^[^ ^12^, ^13^ ^]^ it has aroused extensive attention and research interest from scholars worldwide.^[^ ^14^, ^15^, ^16^, ^17^ ^]^ HEAs contain at least five kinds of elements, and the content of each element is in the range of 5–35% (atomic percent, at%).^[^ ^12^ ^]^ There are no major and minor elements, solvents, and solutes among the elements.^[^ ^12^, ^13^, ^18^ ^]^ Although the types and contents of the elements in HEAs deviate from the Gibbs Phase Rule, the formation of harmful intermetallic compounds can be restrained.^[^ ^19^ ^]^ For example, the representative CrCoFeMnNi equiatomic Cantor alloy^[^ ^13^ ^]^ and CoCrCuFeNi HEA^[^ ^20^ ^]^ possess single phases with faced‐centered‐cubic (FCC) structures. Zhang et al.^[^ ^15^ ^]^ developed CoCrFeNi HEAs with the addition of Al to form a HEA system with a body‐centered‐cubic (BCC) structure. With the increase in the Al content, the alloy phase changed from a single‐phase FCC → two‐phase FCC + BCC → single‐phase BCC structures. The HEA systems, which are composed of refractory elements, tend to form a single‐phase BCC structure, such as TaNbHfZrTi, TaWNbVMo, etc..^[^ ^16^, ^17^ ^]^ The hexagonal‐close‐packed (HCP) phase in the HEA systems can be obtained by composing lanthanides elements with an HCP structure, such as YGdTbDyLu, HoDyYGdTb, DyGdLuTbY, etc.^[^ ^21^, ^22^ ^]^

**Figure 1:**
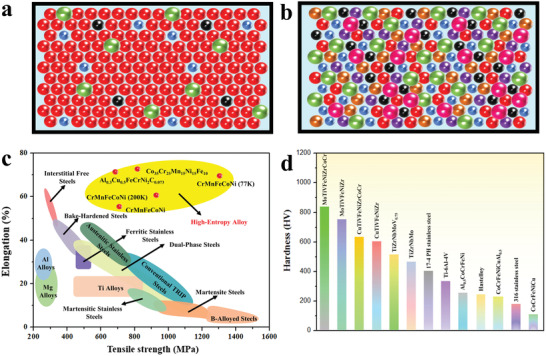
Comparison of the atomic distribution, strength, and hardness between the traditional alloys and HEAs. a) Schematic atomic distribution in traditional alloys, forming dilute solid solutions. A small number of solute atoms occupy the lattice sites and are surrounded by a large number of solvent atoms. b) Schematic atomic distribution in HEAs, composing of multicomponents. Atoms with different atomic radii occupy lattice sites randomly, resulting in serious lattice distortion. c) The Ashby diagram showing the strength comparison between HEAs and conventional alloys. Normally HEAs have high elongation but low strength. d) Hardness of HEAs, 17‐4 PH stainless steel, Hastelloy, and 316 stainless steel. It can be seen that the hardness of the CoCrFeNiCu HEA with a single‐phase FCC structure is very low (< 200 HV), while the MoTiVFeNiZrCoCr HEA with a BCC structure has a very high hardness value (> 800 HV).

In general, HEAs contain many types of elements, and the molar ratios of various components approach equivalent. The characteristics of multiple principal elements make HEAs possess the four core effects: a high mixing entropy, severe lattice distortion, slow diffusion, and cocktail effect.^[^ ^12^, ^23^, ^24^, ^25^ ^]^ The reason why an HEA can form a simple phase rather than a complex one is generally believed to be related to the high‐entropy effect in thermodynamics.^[^ ^12^ ^]^ According to the Boltzmann thermodynamic statistical principle, the configuration entropy of the system, Δ*S*_conf_, is expressed asfollows:^[^ ^12^ ^]^

(1) $$\Delta S_{\mathrm{conf}}=k\;\ln\omega$$

where *k =* 1.38054 × 10^23^ J K^−1^ is the Boltzmann constant, and *ω* is the thermodynamic probability representing the total number of microscopic states contained in the macroscopic state. For equi‐atomic multicomponent‐disordered solid solutions, the configuration entropy of the system with the number of components, *n*, can be expressed as follows:

(2) $$\Delta S_{\mathrm{conf}}=-R\sum_{i=1}^{n}c_{i}\ln c_{i}=R\ln n$$

where *R* = 8.314 J (K mol)^−1^ is the gas constant, and *c_i_* is the mole fraction of the *i* component. When the multicomponent alloy is with an equiatomic ratio,$\;\mathrm{ci}\;=\;\frac{1}{n}$. According to Richards′ law,^[^ ^26^ ^]^ the entropy change of most metals in the melting process is empirically equal to *R* at the melting point. The configuration entropy of equimolar alloys containing three elements has reached 1.1 *R*, which is larger than that of the alloys at the melting point.^[^ ^12^ ^]^ Considering the other positive contributions of the vibrational entropy,^[^ ^27^ ^]^ magnetic entropy,^[^ ^28^ ^]^ and electron entropy,^[^ ^29^ ^]^ the mixing entropy (Δ*S*_mix_) change of an equimolar alloy is even higher than the calculated value.^[^ ^26^ ^]^ The entropy value affects the chaos of the system. The increase in the number of principal elements of an alloy influences mainly the configuration entropy while the vibrational entropy, magnetic entropy, and electron entropy are not dependent on the number of principal alloying elements. Therefore, in order to avoid the difficulty of calculations, the configuration entropy, Δ*S*_conf_, is generally regarded as its mixed entropy, Δ*S*_mix_.^[^ ^12^ ^]^ When various elements are mixed in an equal atomic ratio, Δ*S*_mix_ = *R* ln5 is considered to be a necessary requirement to resist the strong bonding force of atoms at high temperatures.^[^ ^12^ ^]^ Thus, it is considered that five elements are the prerequisites to form high mixing entropy.^[^ ^12^ ^]^ According to the Gibbs‐free energy theory, to keep the stability of the system, the mixture enthalpy and entropy correlate each other, and the mixture entropy plays a leading role in minimizing the Gibbs free energy of the system at high temperatures. Therefore, in the random solution state, the mixing entropy of the HEAs greatly expands the dissolution range of the end‐to‐end solid solution or intermetallic compound, thus forming a simple solid solution. Structurally, HEAs exhibit severe lattice distortion.^[^ ^23^, ^30^ ^]^ Since there is no large difference in the content of each element in the HEA system, there is no difference in solvents and solutes. Therefore, they have the same possibility to occupy the lattice site. The difference in atomic radii between alloy elements causes severe lattice distortion in HEAs,^[^ ^30^, ^31^, ^32^, ^33^, ^34^ ^]^ as shown in **Figure** **1**a,b.

In dynamics, the diffusion rate of the elements forming precipitates in HEAs is slow, directly affecting the coarsening and stability of the precipitates.^[^ ^35^, ^36^, ^37^, ^38^ ^]^ In the Ni_2_CoCrFeNb_0.15_ HEA,^[^ ^35^ ^]^ the size of D0_22_‐Ni_3_Nb precipitates is only ≈15 nm after aging at 650 °C for 80 h. It is well known that D0_22_ is a metastable phase, although the aging time lasts up to 120 h, the size of precipitates is only ≈35 nm without an obvious change in the type of precipitates. L1_2_ and B2 precipitates developed from an FCC and BCC matrix, respectively, after the aging treatment at 923 K in the dual‐phase AlNi_2_Co_2_Fe_1.5_Cr_1.5_ HEA.^[^ ^36^ ^]^ After 213 h, the two kinds of precipitates can still maintain the nanosizes of 57 and 26 nm, respectively. In the Ni‐30Co‐13Fe‐15Cr‐6Al‐6Ti‐0.1B (at%) HEA aged at 800 °C for 720 h,^[^ ^37^ ^]^ although the average size of the L1_2_ precipitate is 93.7 nm, the coarsening rate of L1_2_ precipitates are relatively lower than those in Ni and Co based alloys.^[^ ^39^, ^40^, ^41^ ^]^ The Al_0.2_Co_1.5_CrFeNi_1.5_Ti_0.3_ HEA was aged at 800 °C.^[^ ^38^ ^]^ The average particle size of precipitates increased only 6 nm (from 45 nm after aging for 50 h to 51 nm following aging for 100 h), which represented the slow coarsening of precipitates. The slow diffusion rate of elements in HEAs benefits significantly to the formation of nanoprecipitates and their stability in HEAs.^[^ ^42^, ^43^ ^]^ Upon heating, the change in the Gibb free energy of a system is determined by the changes in the enthalpy, Δ*H*, and entropy, Δ*S*, as follows:

(3) ΔG=ΔH−TΔS

where *T* is the temperature. With the decrease in temperature, the effect of mixing entropy is weakened. From Equation (3), the dominant position of the mixing entropy and enthalpy will intersect at a certain temperature. Below this temperature, intermetallic compounds are expected to be formed, while above this temperature, the alloy keeps a solid solution.

Similar to the traditional alloys, the formation of precipitates in HEAs includes the nucleation and growth. Since nucleation is usually faster than growth, growth is a rate‐limiting step. The growth rates of these precipitates depend on the diffusion of the constituent elements. The diffusivity of elements in HEAs can be expressed as:^[^ ^44^ ^]^

(4) $$D_{s}=D_{0}\exp\left(-Q_{s}/RT\right)$$

where *Q*_s_ is the activation energy of the rate‐controlled diffusion. Assuming that *λ* is half of the average distance between particles, the diffusion distance required for particle growth should satisfy:^[^ ^44^ ^]^

(5) $${\left(D_{s}t\right)}^{1/2\;}\geq \lambda$$

This feature can be converted to:

(6) $$Q_{s}/RT\leq \ln\left(D_{0}t/\lambda^{2}\right)$$

where *t* is the duration of the growth. From Equation (6), it is obvious that there is a corresponding *D*_s_, or more specifically, a temperature at which the diffusion distance is *λ* for a given period. The temperature is labeled as *T*_c_, which is obtained from the above equation:

(7) $$T_{c}=Q_{s}{\left[\ln\left(D_{0}t/\lambda^{2}\right)\right]}^{-1}/R$$

It is reported that HEAs owe heterogeneities from the atomic to nanoscale,^[^ ^45^ ^]^ which leads to the complexity of the atomic distribution in the alloys. This trend may provide more nucleation sites for the precipitation of particles, contributing to the formation of a high number density of nanoscale precipitates. The growth of precipitates can be influenced by the diffusion of elements in HEAs.

For the cocktail effect, HEAs can be regarded as an atomic‐scale composite because it contains many components. Therefore, in addition to the indirect influence of various elements on the microstructure, the basic characteristics of elements and their interaction make the HEAs present a composite effect, namely the “cocktail effect”. In general, it can be understood as a multi‐component composite effect from the atomic to micro scale.^[^ ^25^ ^]^ Currently, HEAs have been extended to four elements or non‐equimolar systems.^[^ ^15^, ^16^, ^17^, ^46^, ^47^, ^48^ ^]^

Compared with the traditional alloys [such as Al alloys,^[^ ^49^ ^]^ Mg alloys,^[^ ^50^ ^]^ Ti alloys,^[^ ^51^ ^]^ interstitial free steels,^[^ ^52^, ^53^ ^]^ bake‐hardened steels,^[^ ^54^ ^]^ austenitic stainless steels,^[^ ^55^, ^56^ ^]^ ferritic stainless steels,^[^ ^57^ ^]^ martensitic stainless steels,^[^ ^58^ ^]^ dual‐phase steels,^[^ ^59^, ^60^ ^]^ and conventional TRIP (transformation‐induced plasticity) steels,^[^ ^61^ ^]^ martensite steels,^[^ ^62^, ^63^ ^]^ and B‐alloyed (boron‐alloyed) steels^[^ ^64^, ^65^ ^]^], the HEAs generally possess a higher hardness, wear resistance, corrosion resistance, and strength. Figure 1c shows the strengths for HEAs along with some other conventional alloys. The Al_1.5_CrFeCoNiCu HEA reaches a hardness of around 500 HV.^[^ ^12^ ^]^ The TiVCrAlSi HEA coatings by laser cladding possesses a hardness up to 800 HV.^[^ ^66^ ^]^ Such a high hardness is mainly due to the contribution of the BCC structure of the matrix. It is known that the FCC phase is generally soft with good ductility, while the BCC phase is hard with less ductility. Figure 1d presents the comparison of the hardness of some representative HEAs along with some other alloys. It can be seen that the hardness of the CoCrFeNiCu HEA with a single‐phase FCC structure is very low (< 200 HV), while the MoTiVFeNiZrCoCr HEA with a BCC structure has a very high hardness value (> 800 HV).

Wu et al.^[^ ^67^ ^]^ studied the effects of Al content on the crystal structures and the hardness of the Al_x_CoCrCuFeNi HEA. They found that the crystal structure changed from the FCC to BCC structure with the increase in the Al content, leading to a significant increase in hardness and wear resistance. Chou et al.^[^ ^68^ ^]^ found that the addition of Mo to the Co_1.5_CrFeNi_1.5_Ti_0.5_Mo_x_ HEA can effectively improve the pitting‐corrosion resistance in a NaCl solution. The passivation film formed by the alloy has self‐healing effects. Luo et al.^[^ ^69^ ^]^ compared the corrosion behavior of an equiatomic CoCrFeMnNi HEA with a 304 L stainless steel in a sulfuric acid solution. They found that both of them had good ability to form a passivation film, but there was no obvious selective dissolution of metal elements during the passivation process on the surface of the equiatomic HEA, and the thickness of the passivation film was greater than that on the 304 L stainless steel. It is found that the Cr content in the passivation film of the equiatomic HEA was much lower than that in the 304 L stainless steel passivation film.

Precipitation strengthening can effectively improve the strengths of the alloys. For instance, He et al.^[^ ^70^ ^]^ developed the (FeCoNiCr)_94_Ti_2_Al_4_ HEA by adding Al and Ti in the FeCoCrNi system. The yield strength of this alloy is ≈1005 MPa, and the tensile strength is up to ≈1,273 MPa with an elongation of ≈17%. The Ni_2_CoCrFeNb_0.15_ HEA designed by He et al.^[^ ^35^ ^]^ possesses a yield strength of ≈ 954 MPa and tensile strength of ≈1,230 MPa with an elongation of 27%. These nanoprecipitate‐strengthened HEAs have a high strength while maintaining a decent ductility due to the slow diffusion in HEAs.^[^ ^35^, ^70^ ^]^ Therefore, nanoprecipitate strengthening is considered as a very promising method to improve the comprehensive mechanical properties of HEAs. However, at present, the influence of the size and type of the nanoprecipitates on the strengthening effect in HEAs is still not clear. The design method and precipitation conditions of the nanoprecipitates are relatively vague.

In this paper, we provide an outlook on the strengthening methods in HEAs briefly first. The focus is mainly placed on the development of nanoscale precipitation‐strengthened HEAs. The formation of precipitates in HEAs and their effects on the mechanical properties are addressed and discussed. Finally, we summarize the design method and the nanoprecipitates‐controlling strategy in HEAs. The present work will provide a useful guide for the future research and design of nanoprecipitate‐strengthened HEAs.

## 2 Strengthening Methods in HEAs

**Figure 2:**
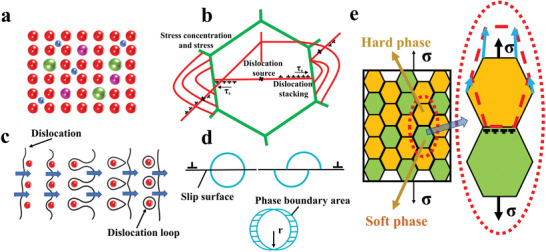
Strengthening mechanisms of HEAs. a) Solid‐solution strengthening. The atoms dissolved in the matrix cause lattice distortion, which increases the resistance of dislocation movement and makes the slip difficult to proceed, thus increasing the strength of the alloy. (The blue ball represents the interstitial atom; the pink ball denotes the replacement atom with the similar size as the matrix atom; the green ball represents the replacement atom with a larger radius.) b) Dislocations are blocked at grain boundaries. Because of the different grain orientations on both sides of the grain boundary, the slip resistance near the grain boundary is increased. Therefore, the slip band cannot directly enter the other side of the grain. c) Dislocation‐bypass mechanism. The moving dislocations are blocked and bent in front of the precipitates. The dislocation loops form, and dislocations proliferate and move forward. d) Dislocation‐cutting mechanism. Coherency strengthening, modulus strengthening, order strengthening, etc. are produced in the process of dislocations cutting through the precipitates. e) Dual‐phase strengthening. In the alloy with a dual‐phase structure, the soft phase is elongated, providing the deformation ability, as shown in the enlarged view. While the hard phase blocks the movement of dislocations to provide strength.

Most of the strengthening methods applied in traditional alloys have been tried in HEAs, including solid‐solution strengthening,^[^ ^71^, ^72^, ^73^ ^]^ grain‐refinement strengthening,^[^ ^74^, ^75^, ^76^ ^]^ dual‐phase strengthening,^[^ ^77^, ^78^ ^]^ phase‐transformation strengthening,^[^ ^79^, ^80^ ^]^ and precipitation strengthening.^[^ ^81^, ^82^, ^83^ ^]^ The grain‐refinement‐strengthening mechanism is summarized in **Figure** **2**a. The properties of HEAs can be tuned by changing the grain size. For example, Schuh et al.^[^ ^74^ ^]^ prepared an equiatomic CoCrFeMnNi HEA with a grain size of ≈ 50 nm through severe plastic deformation. This nano‐grained HEA possesses a high strength of ≈1950 MPa, and a hardness of ≈ 520 HV. The studies on the equiatomic FCC CoCrFeMnNi HEA^[^ ^75^ ^]^ with grain sizes from 4 to 160 µm show that the yield strength obviously increases with the decrease in grain size. According to the Hall–Pitch relationship, the smaller the average grain size is, the higher the yield strength is, which is also applicable to HEAs. In polycrystalline metallic materials, most grain boundaries belong to large‐angle grain boundaries with the adjacent grains possessing different orientation relations. When the dislocations slip to the grain boundary, they will be hindered and cannot directly propagate to the adjacent grains, resulting in the dislocation accumulation within the grains. A small grain size with large and zigzag grain‐boundary areas can promote the plastic deformation under an external force to occur within more grains. This trend will reduce the stress concentration and hinder the crack propagation, improving the strengths and ductility of the materials.

Dual‐phase strengthening was also introduced in the design of HEAs. Moon et al.^[^ ^84^ ^]^ and Son et al.^[^ ^85^ ^]^ successfully designed the HEAs with a dual‐phase structure, using the design strategy of the immiscibility gap in the binary phase diagram. The alloying elements with different crystal structures and strong immiscibility were selected to design dual‐phase alloys.^[^ ^84^ ^]^ For example, Al_x_(CuFeMn)_100‐_ *_x_* (*x* = 0, 7.5, and 15 at%) alloys were developed, using the immiscible nature of Cu–Fe alloys.^[^ ^84^ ^]^ The microstructures of the alloys showed phase separation into Cu‐rich and Fe‐rich regions. The existence of two phases can provide many interfaces between the mechanically‐incompatible domains, which strengthens the alloy through the superposition of solution strengthening and hetero‐deformation‐induced (HDI) strengthening.^[^ ^84^ ^]^ Zhang et al.^[^ ^77^ ^]^ developed a CoCrFeNiCuAl HEA with a FCC + BCC dual‐phase structure. The compressive strength and plastic strain are 1.82 GPa and 20.7%, respectively. The dual‐phase strengthening mechanism is shown in Figure 2e. The as‐cast Al_0.3_CrFe_1.5_MnNi_0.5_ alloy^[^ ^79^ ^]^ is composed of the dendrite BCC and interdendritic FCC phases. Upon annealing, the dendrite BCC matrix changes to a sigma phase without changing the composition, and the dendrite hardness is increased from 386 to 1045 HV. This phase transition is not a general precipitation‐hardening reaction, but a non‐diffusion phase transition from the BCC to tetragonal phase. By adjusting the composition, the dual‐phase structure in HEAs can be realized relatively easily. The effective coordination between the two phases can improve the comprehensive mechanical properties of the materials.

Solid‐solution and precipitation strengthening play important roles in developing novel HEAs. The corresponding strengthening mechanisms are presented in Figures 2b–d. Gao et al.^[^ ^81^ ^]^ designed an equiatomic CrMnFeCoNi HEA with additions of 0.8 at% Nb and C. The additions of 0.8 at% Nb and C stimulated the formation of nanoscale NbC particles inside the grains. The micron‐scale carbides (NbC and M_23_C_6_) were observed at grain boundaries. After microalloying, its yield strength and ultimate tensile strength were significantly increased from 353 and 635 MPa to 732 and 911 MPa, respectively, while maintaining a high elongation of 32%. The strength improvement mainly came from the grain refinement and precipitation strengthening by the Orowan mechanism.^[^ ^81^ ^]^ Zhang et al.^[^ ^82^ ^]^ studied the precipitation behavior and mechanical properties of the aging carbon‐containing Al_0.3_Cu_0.5_CrFeNi_2_ HEA. Compared with the solid‐solution samples, the yield and ultimate tensile strengths of the aged samples at 900 °C were increased by 193 and 415 MPa, respectively, while the elongation was maintained as high as 88%. After aging at 700 and 550 °C, the nanoscale L1_2_ phase appeared in the alloy. The Cu‐rich phase was only found in alloys aged at 700 °C. After 700 °C aging, the strength reached 904 MPa due to the synergistic‐precipitation strengthening of the coherent M_23_C_6_ carbide at the grain boundaries and the L1_2_ and Cu‐rich particles inside the grains. Liu et al.^[^ ^83^ ^]^ synthesized a series of CoCrFeNiNb_x_ HEAs to study the alloying effect of Nb on the structures and tensile properties. They found that the addition of Nb changed the original phase structure, leading to the formation of the ordered Nb‐rich Laves phase embedded in the FCC solid‐solution matrix, contributing to the great strength increase.

Based on the above comparison of the HEA‐strengthening methods, it is found that precipitation strengthening is an effective method. Recently, some maraging steels and HEAs were successfully developed with high strength while maintaining good plasticity. For example, Peng et al.^[^ ^86^ ^]^ found that nanoscale precipitates as a sustainable dislocation source can improve the ductility and strength simultaneously. In the (NiCoFe)_86_Al_7_Ti_7_ alloy strengthened by nanoscale precipitates,^[^ ^87^ ^]^ the obvious dislocation activity and deformation‐induced microbands can completely eliminate the plastic instability. In most cases, however, precipitation strengthening increases the strength of the alloy but reduces the plasticity, which is the “strength‐plasticity” trade‐off. This trade‐off is still the primary problem faced by precipitate‐strengthened alloys, which is largely affected by the size and characteristics of the precipitates.^[^ ^38^, ^88^ ^]^

As we know, when the size of precipitates is large, the strengthening mechanism can be attributed to the Orowan bypass.^[^ ^35^ ^]^ Although these large‐sized precipitates in HEAs strengthened by the Orowan‐bypass mechanism with a good strengthening effect, the ductility of the alloy is seriously reduced at the same time.^[^ ^35^ ^]^ On the other hand, the hard and brittle nature of the precipitates themselves also play a negative role, leading to the early arrival of the alloy fracture under load and a decrease in the ductility.^[^ ^78^, ^89^, ^90^ ^]^ In order to improve the strengths of the HEAs while maintaining high ductility, one can design the fine nanoscale precipitates, possessing a coherent relationship with the matrix. This trend enables HEAs to obtain an excellent combination of strength and plasticity.^[^ ^91^, ^92^, ^93^ ^]^

## 3 Nanoprecipitates

**Figure 3:**
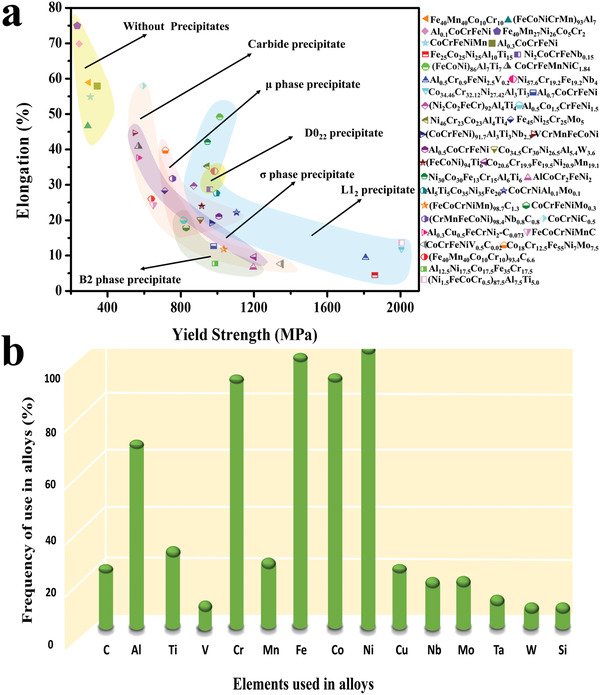
The types of nanoprecipitates and their contributions to strength, and the frequency of elements used in nanoprecipitate‐strengthened HEAs. a) The nanoprecipitates mainly include the carbide precipitate, µ‐phase precipitate, D0_22_ precipitate, *σ* precipitate, L1_2_ precipitate, and B2 precipitate. Among them, L1_2_ and D0_22_ precipitates can effectively strengthen the alloy while maintaining good plasticity. Carbide precipitates can keep the plasticity of the alloy, but it has little contribution to the strength. While µ, *σ*, and B2 phases generally have a negative effect on the plasticity of the alloys. b) In nanoprecipitate‐strengthened HEAs, the use frequency of Cu, Mn, Al, Co, and other elements has changed (the number of samples is 60). It is mainly due to the negative effect of Mn and Cu on the alloy during aging, while Al and Co are widely used as forming and stabilizing elements of the nanoprecipitates.

Due to the limitations of the solute‐element solid solubility, aging response, and other factors, either small‐sized precipitates with a high number density controlled by the nucleation rate or large‐sized precipitates with a low number density caused by the Oswald ripening can form under traditional aging or service conditions. This aging response mechanism often results in high plasticity but a low strength, or a high strength and a sharp decrease in plasticity. Over‐aging can also cause both the strength and plasticity to decrease simultaneously. However, compared with traditional alloys, the unique slow diffusion effect in HEAs will affect the growth rate of the precipitates during the heat treatment, benefiting the formation of small‐sized particles. At present, the small‐sized nanoprecipitates in HEAs mainly include the M_23_C_6_ carbide,^[^ ^94^, ^95^, ^96^, ^97^, ^98^ ^]^ L1_2_ structure *γ*′ phase,^[^ ^99^, ^100^, ^101^, ^102^, ^103^, ^104^, ^105^ ^]^ D0_22_ structure *γ*″ phase,^[^ ^106^, ^107^, ^108^, ^109^, ^110^ ^]^ B2‐phase precipitate,^[^ ^111^, ^112^ ^]^ and some hard *σ* ^[^ ^113^, ^114^, ^115^, ^116^ ^]^ and µ^[^ ^117^, ^118^ ^]^ intermetallic compounds. **Figure** **3**a shows the comprehensive mechanical properties of different kinds of nanoprecipitate‐strengthened HEAs. Next, we will introduce them one by one.

### 3.1 Elemental Composition

Elements in HEAs are mainly concentrated from No. 24–29, in the fourth period of the periodic table, namely Cr, Mn, Fe, Co, Ni, and Cu. The frequency of the use for these six elements is the highest so far.^[^ ^16^ ^]^ The frequencies of elements used in nanoprecipitate‐strengthened HEAs are summarized in Figure 3b. It is found that Ni, Fe, Cr, Co, and Al are the most widely‐used elements in nanoprecipitate‐strengthened HEAs. Since Yeh et al.^[^ ^12^ ^]^ and Cantor et al.^[^ ^13^ ^]^ developed the FCC solid‐solution‐structured HEAs of CoCrCuFeNi and CoCrFeMnNi, respectively, solid solubility among elements was revealed as the most important factor that determines the formation of a FCC single‐phase solid solution.^[^ ^119^, ^120^, ^121^ ^]^

Among the six elements mentioned above, Cu has a larger mixing enthalpy with other elements. Hence, Cu is more likely to precipitate independently in the form of clusters without combining with other elements.^[^ ^88^, ^122^ ^]^ Mn is very active, diffuses quickly, and easily forms oxides, which will seriously reduce the stability of the structure and be very unfavorable when the alloy is treated at high temperatures. Relevant research showed that both quaternary FeCoNiCr and quinary FeCoNiCrMn HEAs have a single FCC solid‐solution structure. Both alloys exhibit the same tensile mechanical behavior in the temperature range of 73–1273 K.^[^ ^123^ ^]^ Therefore, in some studies,^[^ ^124^, ^125^, ^126^, ^127^, ^128^ ^]^ Cu and Mn were completely removed from the selection of matrix‐phase elements of HEAs, or the content of their additions was strictly controlled, or they were added as the high‐strength phase rather than matrix‐phase constituent elements. Co plays an important role in HEAs. On the one hand, Co can be used as a constituent element of the matrix phase. On the other hand, it is also beneficial to the formation of some precipitates. For example, Co is a stabilizer of the *γ*′ precipitates.^[^ ^129^ ^]^ The small incorporation of Co in *γ*′ can also improve the ductility of HEAs.^[^ ^87^ ^]^ However, due to the cost of Co, the cobalt‐free HEAs^[^ ^130^, ^131^, ^132^, ^133^, ^134^ ^]^ have been investigated intensively on the premise without affecting the mechanical properties.

In some HEAs strengthened by nanoscale precipitates, in order to increase the volume fraction of the precipitates to achieve a higher strength, the composition of the matrix phase is usually designed to be simpler, such as the ternary Ni‐Fe‐Co‐based HEAs.^[^ ^87^, ^93^, ^135^ ^]^ Basically, the HEA matrix with nanoscale precipitates is mostly an FCC or dual‐phase structure to ensure the good ductility. Thus, the content of each element in HEAs is no longer constrained to an equiatomic ratio, but adjusted according to the specific condition of the alloys.

### 3.2 Types of Nanoprecipitates

#### 3.2.1 Nanocarbides

The precipitation strengthening of carbides is widely used in traditional steels. Although they are all committed to the low carbonization of steels, the strengthening effect of carbides still provides ideas for alloy strengthening. In HEAs, carbides, specifically nanocarbides, can be applied to improve mechanical properties.^[^ ^81^, ^82^, ^136^, ^137^, ^138^ ^]^

**Figure 4:**
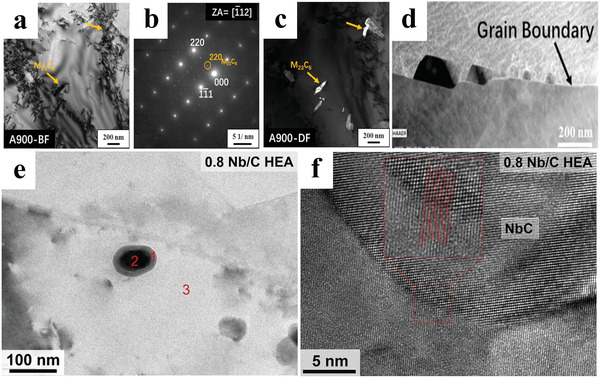
The formation of nanoscale carbides. Al_0.3_Cu_0.5_FeCrNi_2_ HEA aged at 900 °C: a) M_23_C_6_ precipitates in the matrix. b) diffraction pattern with the area axis (ZA). c) Dark‐field image taken with the spot (220)‐M_23_C_6_ in (b). d) Grain‐boundary precipitation. Microstructures of (CrMnFeCoNi)_98.4_Nb_0.8_C_0.8_ HEAs: e) TEM BF image showing the core‐shell structure. f) High resolution transmission electron microscope (HRTEM) image presenting the coherence between the matrix and NbC particles. a‐d) Reproduced with permission.^82^ Copyright 2019, Elsevier. e,f) Reproduced with permission.^81^ Copyright 2019, Elsevier.

M_23_C_6_ is the main form of carbide strengthening. In traditional steels, its size is relatively large in general (at the micron level), but in the carbon‐containing HEA, the size of M_23_C_6_ can be maintained at the nanoscale level. This kind of nanolevel carbides can effectively strengthen the alloy and maintain good ductility. By introducing nanoscale M_23_C_6_ carbides, the yield and ultimate tensile strengths of the Al_0.3_Cu_0.5_FeCrNi_2_ HEA can be improved by 193 and 415 MPa, respectively, as compared with the solid‐solution state, while the elongation still keeps as high as 88%.^[^ ^82^ ^]^ The carbides can form either inside the grains (**Figures** **4**a–c) or at grain boundaries (Figure 4d), depending on the matrix phase, carbon content, and heat‐treatment process.^[^ ^82^ ^]^ When 1.3 at% C was added to the equiatomic FeCoCrNiMn HEA with the annealing temperature of 700 °C, M_23_C_6_ with an average size of 47 nm precipitated uniformly in the recrystallized grains.^[^ ^137^ ^]^ In the Al_0.3_Cu_0.5_CrFeNi_2_ HEA systems, it is found that the carbon content of 0.073 at% is sufficient to promote the formation of the M_23_C_6_ phase, which tends to precipitate at grain boundaries in a needle shape. For M_23_C_6_‐carbide particles, when they have an incoherent relationship with the matrix, the alloys can be strengthened mainly by the Orowan dislocation‐bypass mechanism. However, when they have a coherent relationship with the matrix, such as M_23_C_6_ particles deposited on the Al_0.3_Cu_0.5_CrFeNi_2_ HEA grain boundaries, although their sizes are slightly larger, ductility will not be deteriorated due to the activation of the dislocation‐shearing mechanism.^[^ ^137^ ^]^

In addition to M_23_C_6_, Gao et al. ^[^ ^81^ ^]^ found that nanoscale NbC particles (with an average size of 42 nm) can form in the grains of the CrMnFeCoNi HEA with the additions of small amounts of Nb and C. The fine NbC particles in the HEA have a core‐shell structure, as shown in Figures 4e,f. The core is rich in Nb, and the shell is enriched in Cr and Nb. Due to the extremely‐high hardness and modulus of elasticity of NbC particles, it is difficult for dislocations to cut them even though they have nanoscale sizes and coherent relationships with the matrix.

Besides the precipitation strengthening of these nanoscale carbides, nano‐sized M_23_C_6_ and NbC carbides can fully suppress the growth of the recrystallized grains, contributing to the grain‐refinement strengthening.^[^ ^81^, ^137^ ^]^ Therefore, the carbon‐containing HEAs with dual‐strengthening effects of precipitation strengthening and grain‐refinement strengthening exhibit good mechanical properties.

#### 3.2.2 L1_2_ and D0_22_ Nanoprecipitates

The L1_2_ structured *γ*′ phase (Ni_3_Al) and D0_22_ structured *γ*″ phase (Ni_3_Nb) are mostly used in superalloys.^[^ ^139^, ^140^ ^]^ The presence of these precipitates can effectively strengthen the Ni‐based alloys and obtain a high‐temperature strength greater than that of the Fe‐based^[^ ^141^, ^142^ ^]^ and Co‐based superalloys.^[^ ^143^, ^144^ ^]^ The *γ*′‐strengthening phase in the alloys has a volume fraction of about 20–55%, which can significantly improve the yield strength. Because of the excellent strengthening effect, extensive studies have been carried out to introduce *γ*′ and *γ*′ phases into HEAs to improve mechanical properties.

The size of the *γ*′ phase in HEAs is relatively small, ranging from a few nanometers to tens of nanometers, and the lattice mismatch between the *γ*′ phase and the matrix is also usually small.^[^ ^36^, ^38^, ^145^, ^146^, ^147^ ^]^ Hence, the main strengthening mechanism of the *γ*′ phase is attributed to the order strengthening and dislocation‐cut strengthening. The AlNi_2_Co_2_Fe_1.5_Cr_1.5_ HEA can form nanoscale ellipsoid‐like ordered L1_2_ particles with a diameter of 8 nm in the FCC matrix.^[^ ^36^ ^]^ In the CoCrFeNi(Ni_3_Al)_0.75_,^[^ ^145^ ^]^ the L1_2_ phase was introduced into the matrix by adding Ni_3_Al with a fixed stoichiometric ratio to improve the strength. With the precipitation of nanoscale L1_2_ particles, the yield and tensile strengths can be enhanced to 910 and 1200 MPa, respectively, though the ductility was reduced, as compared with the solid‐solution condition (without nanoscale precipitates).^[^ ^145^ ^]^ The strengthening contribution from the *γ*′ phase strongly depends on its volume fraction. Increasing the Ni content and the Ni/Al ratio is beneficial to the increase in the volume fraction of the *γ*′ phase. The Al_0.5_Cr_0.9_FeNi_2.5_V_0.2_ HEA with 50 at. % Ni and a Ni/Al ratio of 5 has a high volume fraction of L1_2_ phase (>50%).^[^ ^93^ ^]^

**Figure 5:**
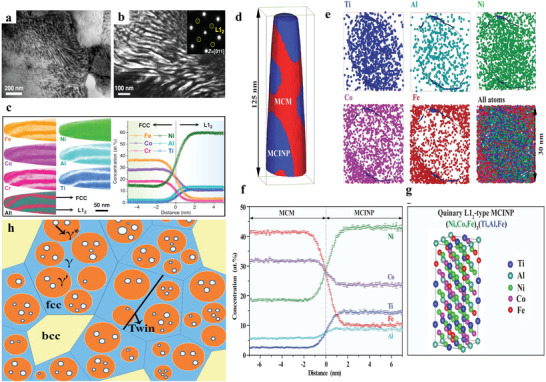
The spatial morphology and multicomponent nature and schematic diagram of three kinds of nanoprecipitates with a complex structure. a) Bright TEM field image shows the morphology of a nanolamellar structure in the (Ni_1.5_FeCoCr_0.5_)_87.5_Al_7.5_Ti_5.0_ HEA. b) Dark‐field image presents that nanolamellae possess an L1_2_ structure. c) APT presents that the nanolamellar structure is mainly composed of Ni_3_(Al,Ti). a‐c) Reproduced with permission.^[^ ^92^ ^]^ Copyright 2020, Nature Publishing Group. d) 3D reconstruction of the atom probe tomography (APT), showing the nanocomposite microstructure in the (FeCoNi)_86_A1_7_Ti_7_ alloy. e) Corresponding 3D APT atom maps exhibit the atomic distribution in a typical L1_2_ precipitate. f) The near‐histogram through the matrix and nanoparticles reveals the multicomponent nature of the MCINPs. g) Sorting the crystal structure and site occupancy of the L1_2_ MCINP by the density functional theory (DFT) calculation of the (FeCoNi)_86_A1_7_Ti_7_ alloy. d‐g) Reproduced with permission.^[^ ^87^ ^]^ Copyright 2018, American Association for the Advancement of Science. h) Schematic diagram of the microstructure consisting of the *γ* matrix grains (blue) and layered intragranular precipitates, namely primary *γ*′ precipitates (orange). The secondary γ* precipitates (white) located in the primary *γ*′ precipitates. Reproduced with permission.^[^ ^135^ ^]^Copyright 2018, American Association for the Advancement of Science.

Ti can substitute the position of Al in the *γ*′(Ni_3_Al) phase, forming a *γ*′ phase with a more complex composition of Ni_3_(Al, Ti). This complex *γ*′ phase can enhance the strengthening effect.^[^ ^129^ ^]^ Thus, the influences of the addition of Ti along with Al on the precipitation of the *γ*′ phase have been investigated extensively.^[^ ^38^, ^46^, ^70^, ^130^, ^148^, ^149^ ^]^ On the other hand, Ti can act as a forming agent for the L1_2_ phase, contributing to the formation of the L1_2_ structure of the Ni_3_(Al, Ti) *γ*′ phase.^[^ ^146^ ^]^ In addition, according to Yang et al.,^[^ ^87^ ^]^ the high content of Al in the alloy will cause the severe environmental embrittlement in air. Replacing Al by Ti and decreasing the Al content can alleviate the environmental embrittlement. In the Al_0.2_Co_1.5_CrFeNi_1.5_Ti_0.3_ HEA,^[^ ^38^ ^]^ nano‐sized spherical coherent and ordered L1_2_ Ni_3_(Al, Ti) precipitates formed after aging at a temperature of 700–1000 °C. After aging for a proper period, the yield and ultimate tensile strengths increased without significantly reducing the ductility. This trend was attributed to the uniformly‐distributed nanoscale coherent precipitates in the coarse‐grained solid solution.^[^ ^38^, ^147^ ^]^ In a recent study,^[^ ^92^ ^]^ the (Ni_1.5_FeCoCr_0.5_)_87.5_Al_7.5_Ti_5.0_ HEA exhibits excellent mechanical properties due to its unique nanolamellar structure. The morphology of a nanolamellar structure is shown in **Figure** **5**a. The ultra‐high strength mainly comes from the lamellar boundary strengthening, whereas the high ductility is related to the progressive work‐hardening mechanism regulated by the nanolamellar structure composed of Ni_3_(Al,Ti). Figures 5b,c present the structure and chemical composition of the nanolamellae. The coherent lamellar boundary facilitates dislocation transmission, thereby eliminating the stress concentration at the boundary. At the same time, the stacking‐fault networks caused by deformation and the associated high‐density Lomer–Cottrell locks enhance the work‐hardening response, resulting in exceptionally‐large tensile ductility.^[^ ^92^ ^]^

The L1_2_‐structured *γ*′ phase is a metastable phase. Under appropriate external conditions or when the composition changes, the metastable L1_2_‐structured *γ*′ phase can change to the stable *η* phase. The *η* phase is a kind of hard intermetallic compound, deteriorating the comprehensive mechanical properties. This transition between the phase structures can be effectively controlled by adjusting the composition and the ratio of Ti and Al. The addition of Ti promotes the formation of L1_2_ and *η* phases in CoCrFeNi.^[^ ^146^ ^]^ The addition of Al can promote the formation of the B2 phase and reduce the types of intermetallic compounds. When the Ti/Al ratio is within 0.7–2, the L1_2_ phase can be obtained.^[^ ^130^, ^146^ ^]^ The B2 phase is apt to form when Ti/Al = 1/3. When Ti/Al = 3, a lath *η* phase and spherical *σ* phase can form along with the L1_2_ phase. Ti can be regarded as the L1_2_ phase‐forming agent, and Al, the L1_2_ phase stabilizer. It is also found that there is no apparent effect of the Ti/Al ratio on the average size of the *γ*′ phase.

The L1_2_ phase possesses an FCC structure, which can produce a small lattice mismatch with the matrix phase that also has the FCC structure, so that it will not cause the serious stress concentration while hindering the dislocation slip. The strengthening effect of the L1_2_ phase is mainly derived from the orderly strengthening caused by the addition of foreign elements. Since the bonding force between atoms of different elements is greater than that between atoms of the same elements, the ordered arrangement of atoms of different types will contribute to a higher strength for the ordered alloy. According to the expression of the atomic ordering (Δ*σ*_os_):^[^ ^70^, ^93^, ^150^, ^151^, ^152^ ^]^

(8) $$\Delta \sigma_{\mathrm{os}}=M0.81\frac{\gamma_{\mathrm{APB}}}{2b}{\left(\frac{3\pi f}{8}\right)}^{1/2}$$

where *M* is the Taylor factor, *b* is the Burgers vector, *γ*_APB_ is the antiphase‐boundary energy of the precipitates, and *f* is the volume fraction of the precipitates. It is obviously that increasing the volume fraction of the precipitates is the simplest and most effective way to improve the orderly strengthening effect, thereby enabling the alloy to exhibit a higher yield strength.

In some cases, higher contents of Al and Ti were designed to increase the volume fraction of *γ*′ precipitates in order to enhance the strengthening effect. Multicomponent intermetallic nanoparticles (MCINPs) were introduced in the (FeCoNi)_86_‐Al_7_Ti_7_ HEA.^[^ ^87^ ^]^ The *γ*′ MCINPs with an L1_2_ structure are distributed inside the grains with a nearly‐spherical shape and size between 30 and 50 nm. As shown in Figures 5d–g,^[^ ^87^ ^]^ a high volume fraction of *γ*′ precipitates up to 50% to 55% can be obtained. The lattice mismatch between the MCINPs and FCC matrix is 0.21%. The alloy possesses a yield strength of >1.0 GP, a tensile strength of ≈1.5 GPa, and an elongation of up to ≈ 50%. Its excellent strength‐plasticity combination is attributed to the great combination of precipitation strengthening and work hardening. Complex nanoscale‐precipitates with core‐shell structures can enhance the mechanical properties significantly. Fu et al. ^[^ ^135^ ^]^ designed an Fe_25_Co_25_Ni_25_Al_10_Ti_15_ HEA with an ultimate tensile strength of ≈ 2.52 GPa and an elongation of 5.2% strengthened by the core‐shell *γ*′ + γ* phases, in which the secondary γ* phase locates in the *γ*′ structure, as presented in Figure 5h. They introduced a high density of complex particles by mechanical alloying (MA). The *γ*′ + γ* complex particles with a diameter of 57 nm are located in the FCC matrix. The volume fraction of the *γ*′ + γ* complex particles is ≈ 51%. In addition, due to the coherent relationship among *γ*, *γ*′, and γ* phases, it is believed that the dislocation‐cutting mechanism is the main strengthening factor.

**Figure 6:**
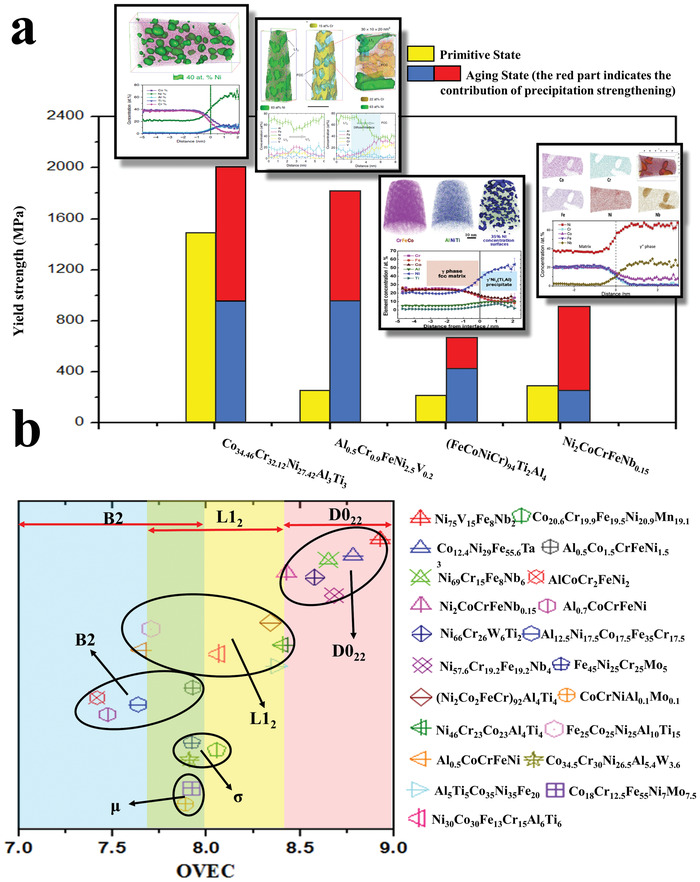
The contribution of L1_2_ and D0_22_ nanoprecipitates to the strength of HEAs, and the effect of overall valence electron concentration (OVEC) on the formation of nanoprecipitates. a) Generally, the contribution of nanoprecipitates can double the strength. The illustrations correspond to the atomic diagrams of different elements and 3D structures of nanoprecipitates.^[^ ^35^, ^46^, ^70^, ^93^ ^]^ b) The effects of OVEC on the formation of precipitates. The OVEC > 8.4 is favorable for the precipitation of D0_22_ nanoprecipitates. The OVEC ranging from 7.7 to 8.4 is preferred for the formation of L1_2_ nanoprecipitates. The OVEC for *σ* and µ phases ranges within 7.8–8.2. The range of OVEC between 7.3 and 8.0 is favorable for the development of B2 nano‐precipitates. a) Images of APT microstructures from left to right: Reproduced with permission.^46^ ^]^ Copyright 2020, Nature Publishing Group. Reproduced with permission.^93^ Copyright 2018, Nature Publishing Group. Reproduced with permission.^70^ ^]^ Copyright 2016, Elsevier. Reproduced with permission.^35^ ^]^ Copyright 2019, Elsevier.

Although increasing the volume fraction of the *γ*′ phase in HEAs can effectively improve the comprehensive mechanical properties, but increasing the volume fraction of the *γ*′ phase will also cause the concentration increase of constituent elements of the *γ*′ phase, such as Al and Ti. This trend may increase the risk of the formation of other intermetallic compounds or cause other processing problems. In Ni‐based superalloys, the D0_22_ superlattice‐structured *γ*″ phase (Ni_3_Nb) is the most widely‐used strengthening phase besides the *γ*′ phase.^[^ ^153^, ^154^ ^]^ According to the strengthening equation of the *γ*′ phase:^[^ ^35^, ^155^ ^]^

(9) $$\Delta \sigma_{\mathrm{coherency}}=1.7MG{\left|\varepsilon \right|}^{3/2}{\left[\frac{h^{2}f\left(1-\beta \right)}{2bR}\right]}^{1/2}$$

(10) $$\Delta \sigma_{\mathrm{ordering}}^{\gamma^{\prime \prime }}=M\left(\frac{\gamma_{\mathrm{APB}}}{2b}\right)\left\{{\left[\frac{4\gamma_{\mathrm{APB}}f}{\pi T}{\left(\frac{\sqrt{6}Rh}{3}\right)}^{1/2}\right]}^{1/2}-\beta f\right\}$$

where *G* is the shear modulus, *b* is the magnitude of the Burgers vector, *f* is the volume fraction of precipitates, *ε* is the tetragonal lattice misfit, *R* is the real diameter of the particles, *h* is the half thickness of the particles, *M* is the Taylor factor, *β* is a constant, *γ*_APB_ is the antiphase‐boundary energy of the *γ*′ phase, and *T* is the line tension. Although the *γ*′ phase has the same FCC structure as the *γ*′ phase, it usually possesses a higher antiphase‐boundary energy and greater crystal‐lattice mismatch. The yield strength of the *γ*″ phase can be dominated by coherency strengthening and ordering strengthening, leading to a better strengthening effect.^[^ ^156^, ^157^ ^]^ In Ni‐based superalloys, the *γ*′ (Ni_3_Nb)‐strengthening phase contributes much more to the strength than the *γ*′ phase below 700 °C. This design strategy has also been applied to the development of nanoprecipitate‐strengthened HEAs. For example, the *γ*′ phase with a coherent D0_22_ superlattice structure can strengthen the Ni_2_CoCrFeNb_0.15_ HEA significantly.^[^ ^35^ ^]^ The composition of the *γ*′ phase in this HEA contains about 7.7% (at%) Co, which is different from the traditional *γ*″ phase of Ni_3_Nb (without Co). Disk‐shapes *γ*′, particles with a small volume fraction (7%) will make the yield strength increase to 954 MPa, and the tensile strength reaches 1230 MPa with an elongation of ≈ 27%. The excellent strengthening effect of the *γ*′ phase is attributed to the ordered and coherent strengthening. The lattice mismatch between the precipitates and the matrix is 0.012, showing a coherent relationship. This small size and coherent *γ*′ precipitates eventually lead to a strengthening mechanism of dislocation cutting. **Figure** **6**a summarizes the contribution of L1_2_ and D0_22_ nanoprecipitates to the strength of HEAs.^[^ ^35^, ^46^, ^70^, ^93^ ^]^

#### 3.2.3 Other Nanoprecipitate‐Strengthened HEAs

Besides L1_2_ and D0_22_ nanoprecipitates, some other nanoscale precipitates are also introduced to strengthen HEAs, such as Cu‐rich nanoclusters, B2‐structured NiAl phases, hard *σ*, and μ intermetallic compounds, etc. The Cu‐rich nanoclusters have been successfully applied in designing Cu‐rich nanoprecipitate‐strengthened steels.^[^ ^158^, ^159^ ^]^ These kinds of nanoclusters are also introduced in the design of HEAs.^[^ ^82^, ^149^, ^160^, ^161^, ^162^ ^]^ Even after the cryogenic (−40 °C) treatment that can suppress the formation of the additional phases, the Cu‐rich nanoclusters with a diameter of 10–15 nm can still be observed in the CoCuFeNiTa_0. 5_ HEA.^[^ ^162^ ^]^ It was found that in the Al_0.3_Cu_0.5_CrFeNi_2_ HEA after aging at 550 °C for 150 h, only an L1_2_ phase emerged while L1_2_ and Cu‐rich phases were both found after aging at 700 °C for 50 h, indicating that the precipitation kinetics of the Cu‐rich phase is possibly slower than that of the L1_2_‐ordered phase.^[^ ^82^ ^]^ In HEAs, only a small amount of Cu can gather together, forming clusters, and most of Cu can still be dissolved in the matrix and become a constituent element of the alloy matrix.^[^ ^163^, ^164^, ^165^, ^166^ ^]^

Liu et al.^[^ ^167^ ^]^ found that the Mo addition in the CoCrFeNi‐based HEA promoted the precipitation of extremely‐hard *σ* and μ intermetallic compounds in the FCC matrix. These particles precipitated from the supersaturated FCC matrix are relatively fine due to the slow diffusion process in the HEAs, and very effective for strengthening the alloys. This kind of precipitates, which are composed of the complex *σ* phase or the fine *σ* and μ intermetallic phases, greatly enhances the strength of the CoCrFeNiMo_0.3_ alloy. The yield strength of the precipitation‐enhanced HEA at room temperature is 816 MPa, the tensile strength is 1.2 GPa, and the elongation is about 19%. In the CoCrFeNiMo_0.85_ HEA,^[^ ^168^ ^]^ the *σ* and secondary μ phases were also observed. After annealing at 900–1000 °C, the *σ* phase can completely transform into the μ phase, leading to a reduction in hardness.

When these small‐sized hard and brittle intermetallic‐compound precipitates and tough precipitates coexist in the alloys, they act as auxiliary phases for strengthening HEAs. The B2, L2_1_, and *σ* phases are generally difficult to nucleate in the grains. They are mostly precipitated on grain boundaries, which limits the growth of the grains and plays a role in contributing to the strength.^[^ ^149^ ^]^ Furthermore, the B2 phase with a BCC structure is easy to precipitate in the HEA matrix of the BCC structure,^[^ ^169^ ^]^ just as the *γ*′ phase with the ordered L1_2_ structure can precipitate in the HEA matrix of the FCC structure, improving the strength of the HEAs.

However, when the metastable phases in alloys are under over‐aging conditions, they will transform into the hard and brittle intermetallic compounds. In this case, in order to obtain better comprehensive mechanical properties, they are also regarded as harmful phases. For example, after aging at 650 °C for 120 h, the *γ*′ phase will transform into a nano‐sized *δ* phase precipitated at grain boundaries.^[^ ^35^ ^]^ In the (FeCoNiCr)_94_Ti_2_Al_4_ HEA after aging at 923 K for 4 h,^[^ ^70^ ^]^ in addition to the *γ*′ phase of the L1_2_ structure, irregular bulk L2_1_ structural Ni_2_AlTi particles with a size of ≈100 nm are also observed, which are non‐coherent with the FCC HEA matrix. The L2_1_‐structure particles have also been found by Yang et al..^[^ ^87^ ^]^ The presence of the *σ* phase was found in the Al_0.3_Cu_0.3_Ti_0.2_CoCrFeNi HEA developed by Gwalani et al..^[^ ^149^ ^]^ The as‐rolled alloy is composed of FCC and L2_1_ phases. However, after aging at 600 °C, ≈34% (volume percent, vol%) of L1_2_ phases and ≈10% (vol%) of *σ* phases also appear in the alloy. When the aging temperature is increased to 800 °C, there is no change in the phase composition of the alloy, and only the volume fraction changes. This *σ* phase has also been reported in the CoCrFeNi(Ti_x_Al_y_)_0.2_ HEA with Ti/Al = 3.^[^ ^146^ ^]^

In order to better explore the effects of the types and sizes of nanoprecipitates on the strengthening effect of HEAs, we summarize the precipitation temperatures, types of precipitates, yield strengths, tensile strengths, and ductility of some representative nanoprecipitate‐strengthened HEAs, as listed in **Table** **1**. The strengthening of *γ*′ or *γ*′ precipitates in the FCC structure is not strong though the plasticity can be maintained well during precipitation strengthening due to the low lattice mismatch between the FCC matrix and precipitates. Thus, the volume fraction must be increased to enhance the strength. The carbides, Cu‐rich nanoprecipitates, *σ*, µ, and B2 phases can effectively hinder the dislocation movement due to the non‐coherent relationship with the matrix, resulting in a high strength. The size of the nanoprecipitates and the alloy systems also play a great role in the strengthening effect.

**Table 1:** Precipitation temperatures, types of precipitates, and mechanical properties of nanoprecipitate‐strengthened HEAs

Alloy	Temperature [°C]	Precipitates	Yield strength [MPa]	Tensile strength [MPa]	Ductility [%]	References
FeCoCrNiMn‐1.3C	700	M_23_C_6_	1040	1112	12	^[^ ^137^ ^]#^
Al_0.3_Cu_0.5_FeCrNi_2_‐C_0.073_	900	M_23_C_6_	255	584	88	^[^ ^82^ ^]#^
(CrMnFeCoNi)_98.4_Nb_0.8_C_0.8_	800	M_23_C_6_, NbC	732	911	32	^[^ ^81^ ^]#^
Al_0.3_Cu_0.5_FeCrNi_2_‐C_0.073_	550	M_23_C_6_, *γ*′	350	642	73	^[^ ^82^ ^]#^
Al_0.3_Cu_0.5_FeCrNi_2_‐C_0.073_	700	M_23_C_6_, *γ*′, Cu–rich	535	904	39	^[^ ^82^ ^]#^
(FeCoNiCr)_94_Ti_2_Al_4_	800	*γ*′	645	1094	39	^[^ ^70^ ^]#^
(FeCoNiCr)_94_Ti_2_Al_4_	650	*γ*′	1005	1273	17	^[^ ^70^ ^]#^
Ni_46.4_Al_5_Co_5_Cr_21.2_Fe_15_Ti_1.5_Nb_3.1_Mo_2.8_	720 + 620	*γ*′	970	1310	32	^[^ ^148^ ^]#^
(FeCoNi)_86_Al_7_Ti_7_	780	*γ*′	>1000	≈1500	≈50	^[^ ^87^ ^]#^
Al_0.5_Cr_0.9_FeNi_2.5_V_0.2_	600	*γ*′	1810	1905	9–10	^[^ ^93^ ^]#^
Co_34.46_Cr_32.12_Ni_27.42_Al_3_Ti_3_	700	*γ*′	≈2000	≈2200	≈13	^[^ ^46^ ^]#^
CoCrFeNi (Ni_3_Al)_0.75_	700	*γ*′	910	1200	14	^[^ ^145^ ^]#^
Al_0.2_Co_1.5_CrFeNi_1.5_Ti_0.3_	800	*γ*′	750	1160	12	^[^ ^38^ ^]#^
Al_0.2_CrFeCoNi_2_Cu_0.2_	700	*γ*′	719	1048	30.4	^[^ ^161^ ^]#^
(Ni_1.5_FeCoCr_0.5_)_87.5_Al_7.5_Ti_5.0_	600	*γ*′	2026	2118	16	^[^ ^92^ ^]#^
Ni_2_CoCrFeNb_0.15_	650	*γ*″	954	1230	27	^[^ ^35^ ^]#^
Fe_25_Co_25_Ni_25_Al_10_Ti_15_	‐	γ*, *γ*′	≈1860	≈2520	5.2	^[^ ^135^ ^]#^
(FeNi)_67_Cr_15_Mn_10_Al_3_Ti_5_	800	*η*, *γ*′	812	1247	22	^[^ ^130^ ^]#^
Al_0.3_Cu_0.3_Ti _0.2_CoCrFeNi	600	L2_1_, *σ*, *γ*′	820	1100	20	^[^ ^149^ ^]#^
CoCrFeNiMo_0.3_	850	*σ*, *μ*	816	1200	19	^[^ ^167^ ^]#^
AlNi_2_Co_2_Fe_1.5_Cr_1.5_	650	BCC, *γ*′	≈1000	1240	≈13	^[^ ^36^ ^]#^
Al1_2.5_Ni_17.5_Co_17.5_Fe_35_Cr_17.5_	700	BCC/B2	991	1245	8.2	^[^ ^147^ ^]#^
Al_0.7_CoCrFeNi	580	FCC+L1_2_/BCC+B2	990	1400	13	^[^ ^169^ ^]#^

### 3.3 Design of Nanoprecipitates

Although the introduction of nanoprecipitates in HEAs can effectively improve the comprehensive mechanical properties in theory, all the factors, including the type, size, morphology, precipitation location, and conditions, the relationship between the precipitates and matrix, and the mutual transformation of the structure types between the nanoprecipitates, can directly affect the final strengthening effect. Therefore, the design of nanoprecipitates in HEAs is the key to achieve excellent mechanical properties. This section summarizes the design strategy of nanoprecipitates in HEAs, including their compositions and structure design of nanoprecipitates and the precipitation‐temperature conditions.

#### 3.3.1 Design of the Structures of Nanoprecipitates

Ma et al.^[^ ^147^ ^]^ investigated the effects of elements in the Al_2_(Ni, Co, Cr, Fe)_14_ HEA through the cluster formula by systematically changing the content of the transition metal element within the Al_2_M_14_ chemical formula (M stands for different transition metal elements) instead of changing the Al content. In the FCC and BCC solid solutions, the nearest clusters are cubic octahedra with a coordination number of 12 (CN12), and diamond‐shaped dodecahedron with a coordination number of 14 (CN14), respectively. It is also verified that the phase formation and mechanical properties of these Al_2_M_14_ HEAs are closely related to the valence electron concentration (VEC). Thus, the lattice mismatch between the nanoprecipitates and matrix can be optimized to enhance the performance of nanoprecipitate‐strengthened HEAs.

VEC is one of the main factors affecting the structures of nanoprecipitates in HEAs. Liu et al.^[^ ^170^ ^]^ found that the crystal structure of the (Co, Ni, Fe)_3_V alloy changed from the tetragonal (8.75 < VEC) to pure cubic order (VEC < 7.89), and then transformed to an ordered hexagonal structure when 7.89 < VEC < 8.75. Guo et al. also studied the effect of VEC on the matrix phase of HEAs.^[^ ^181^ ^]^ They stated that the FCC phase is stable at a higher VEC (>8), while the body‐centered‐cubic phase is stable at a lower VEC (<6.87) in HEAs. Moreover, He et al.^[^ ^35^ ^]^ summarized the change of crystal structures of typical intermetallic compounds with VEC. Generally, the crystal structure changes from a tetragonal to a hexagonal structure as VEC decreases, and then to a cubic structure, indicating that the main structural‐formation parameters of ordered intermetallic alloys are closely related to the VEC value. Therefore, they proposed a new design strategy based on the OVEC, and designed the *γ*′‐phase precipitates with a coherent D0_22_ superlattice structure, contributing to a strong strengthening effect. The proposed OVEC design strategy shows that when the OVEC values of the HEAs are >8.4, the *γ*′ phase tends to form. Moreover, it is found that the elements from the VB group in the periodic table promote the precipitation of the *γ*′ phase in FCC HEAs. Figure 6b depicts the effect of OVEC on the precipitation of different kinds of nanoprecipitates.

Furthermore, Liang et al.^[^ ^93^ ^]^ designed an Al_0.5_Cr_0.9_FeNi_2.5_V_0.2_ HEA. The alloy‐design strategy in this HEA involves the initial composition and the matrix composition after precipitates separate out from the matrix. This trend is different from the previous HEA‐design concept. According to the previous opinions, only the initial composition was considered rather than addressing whether the final main phase was a high‐entropy solid solution, which may lose the advantages of HEAs. In the CoCrFeNi(Ni_3_Al)*_x_* (*x* = 0.25, 0.5, 0.75, and 1) HEA,^[^ ^145^ ^]^ the *γ*′ phase was introduced by directly adding the L1_2_‐structured Ni_3_Al with a perfect chemical formula ratio. The outstanding advantage of this HEA‐design strategy is that a HEA with excellent comprehensive properties can be obtained by selecting a matrix with excellent properties and combining with an appropriate strengthening phase. This process provides a new strategy for the HEA design.

There are also some reports on the design of layered nanoprecipitates and dual‐phase nanoprecipitates. For example, Fu et al.^[^ ^135^ ^]^ designed a HEA with layered nanoprecipitates for its high strength. Al and Ti tend to form intermetallic phases with Fe, Ni, and Co, because their mutual solubility is very high. Therefore, adding Al and Ti to the FeCoNi alloy is expected to form high‐density intragranular nanoprecipitates. In addition, this HEA also satisfies the empirical conditions of *Ω* ≥ 1.1 and *δ* ≤ 6.6%, where *Ω* is an index that weighs the stability of multi‐component solid solutions ($\Omega =\frac{T_{m}\Delta S_{\mathrm{mix}}}{\left|\Delta H_{\mathrm{mix}}\right|}$,^[^ ^135^ ^]^ where *T*_m_ is the liquidus temperature of alloys, Δ*S*_mix_ is the entropy of mixing, and Δ*H*_mix_ is the enthalpy of mixing), and *δ* represents the difference in the atomic sizes between constituent elements $\left(\delta =\sqrt{\sum_{i=1}^{n}c_{i}{\left(1-r_{i}/r\right)}^{2}}\right)$, where *r_i_* is the atomic radius of the nth element, *c_i_* is the atomic percentage of the nth element, and $r=\sum_{i=1}^{n}c_{i}r_{i}$ is the average atomic radius).^[^ ^171^, ^172^ ^]^ *Ω* infinitely close to the threshold of 1.1 may lead to the formation of secondary precipitates.^[^ ^135^ ^]^ Therefore, according to this concept, the Fe_25_Co_25_Ni_25_Al_10_Ti_15_ HEA with *Ω* = 1.119 and *δ* = 6.277 was designed. The experimental results show that the *γ*′ phase with an L1_2_ structure can be generated in the disordered FCC matrix. In addition, the existence of another phase, γ*, in the *γ*′ phase was also observed. However, in the HEA with a dual‐phase FCC + B2 structure, the ellipsoidal‐ordered L1_2_ precipitates with a diameter of 8 nm were observed in the FCC dendrites, while BCC‐structured precipitates with a diameter around 10 nm were also observed in the B2 dendrites.^[^ ^33^ ^]^ In the Al_0.7_CoCrFeNi HEA,^[^ ^169^ ^]^ Cr‐rich BCC nanoprecipitates were observed in the B2 phase.

#### 3.3.2 Precipitation Temperatures of Nanoprecipitates

Nano‐precipitates in HEAs usually form during aging treatments. Temperature is the key factor determining the nanoscale precipitation and provides the energy required for the nanoscale precipitates. Aging time is also an important parameter, which determines the evolution of the size and number density of nanoprecipitates. Both the aging temperature and time have great influence on the precipitation, morphology, and transformation of nanoprecipitates. The precipitation temperatures of nanoprecipitates in some representative HEAs are summarized in Table 1. For example, He et al.^[^ ^70^ ^]^ employed the proper heat treatment to make the distribution of coherent nanoscale precipitates more dispersed, and observed spherical and lath‐shaped L1_2_ precipitates with different morphologies. In the Al_0.2_CrFeCoNi_2_Cu_0.2_ HEA,^[^ ^161^ ^]^ the L1_2_ phase was present in the alloy after aging at 700 °C for 20 h, while there was no nanoscale precipitation after 1 h at 800 °C. Shafiee et al.^[^ ^148^ ^]^ studied the effects of direct aging and solution aging on the nanoscale precipitates. Direct‐aging samples only consisted of the *γ* and *γ*′ phases, while the re‐precipitation of the Laves phase was detected inside the grains and on the grain boundaries for solution‐aging samples.

It can be seen from Table 1. that the precipitation temperature of the *γ*′ nano‐precipitates is between 600 and 800 °C, which is higher than that of the *γ*′ nano‐precipitates (between 500 and 750 °C). This trend is consistent with the temperature range of these two phases in Ni‐based superalloys. The precipitation temperature of the Cu‐rich nanoprecipitates is around 700 °C, and the precipitation kinetics of the Cu‐rich nanoprecipitates is slower than that of the L1_2_‐ordered phase.^[^ ^82^ ^]^ The precipitation temperature of carbides is much wider (between 550 and 800 °C) and thus more stable.

## 4 Discussion

Although all kinds of precipitates have been introduced in HEAs from the large‐scale to small‐sized ones, and then to more widely‐studied *γ*′ phase with an L1_2_ structure or *γ*′ phase with a D0_22_ structure, the comprehensive mechanical properties obtained at present do not seem to reach full potentials of HEAs. Therefore, it is necessary to explore new nanoprecipitates and new strengthening mechanisms. The new type of nanoprecipitates should exhibit a strength enhancement without scarifying the ductility.

The advanced nanoprecipitate‐strengthened HEA should have the following characteristics of nanoprecipitates: 1) a small lattice mismatch with the matrix. 2) Higher shear modulus and antiphase‐domain‐boundary energy. 3) More stable phase structure and simpler precipitation conditions. 4) Greater number density and smaller size. 5) High thermal stability at elevated temperatures. With a small lattice mismatch between the nanoprecipitates and the matrix, the specific interface energy between the matrix phase and the precipitates can be reduced, which is conducive to maintaining the nanoscale size of the precipitates.^[^ ^46^ ^]^ The high shear modulus and antiphase‐domain‐boundary energy ensure that it is difficult for the dislocation to cut and provide considerable coherent strengthening, modulus strengthening, and ordered strengthening.^[^ ^35^ ^]^ A high number density of nanoprecipitates can be obtained by controlling a small lattice mismatch between the coherent matrix and precipitates, and low driving force of precipitation nucleation. Moreover, a certain amount of defects reserved in the matrix phase can promote the high density precipitation of nanoprecipitates.^[^ ^70^ ^]^

Besides the properties of nanoprecipitates, the behavior of the matrix are also playing an important role in obtaining comprehensive mechanical properties. On the one hand, the coordination between the matrix and precipitates directly affects the strengthening effect of the precipitates. On the other hand, the matrix phase is also the main source of the contribution to strengthening and the main factor affecting the overall plasticity of the alloy. Therefore, the design of the matrix phase should have the following characteristics: 1) Large solid solubility among the elements of the precipitates in the matrix, so as to ensure the continuous supply of components for the precipitation in the aging process. 2) Small lattice mismatch between the precipitates and matrix to stabilize the coordination relationship between the two phases. 3) Good deformation ability to bear the stress concentration caused by precipitation hardening under external loads. 4) Certain strengthening effect, which can be used as the secondary source of the alloy strengthening. Therefore, it is necessary to design the proper composition and structure of the matrix phase.

**Figure 7:**
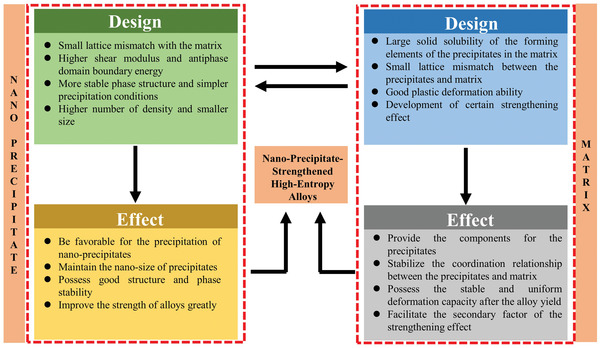
The design concept of the nanoprecipitate‐strengthened HEAs. The design concept involves two aspects: nanoprecipitates and matrix phases. The multi‐component / high‐density nanoprecipitates will contribute more to the strength while maintaining good plasticity. The matrix phase should possess good deformation ability, providing the secondary source of the strengthening effect. The nanoprecipitates and matrix are not isolated from each other, and one should pay more attention to the coordination of these two phases.

In addition to the design criterion of the nanoprecipitates and matrix phase, the following is a further summary of the matrix‐phase composition and structure design, including the twin‐induced and phase‐transformation‐induced plasticity, non‐uniform microstructure design, and short‐range chemical order. The stacking fault energy of the alloy can be effectively reduced by the proper selection and concentration control of alloy elements, which will form deformation twins in the process of deformation, and then cause twin‐induced plastic deformation.^[^ ^80^ ^]^ The deformation‐twin boundaries cannot only hinder the movement of dislocations, but also absorb the dislocations to withstand large plastic deformation, effectively improving the comprehensive mechanical properties.^[^ ^173^, ^174^ ^]^ When the stacking fault energy continues to decrease, phase‐transformation‐induced plastic deformation can occur, and the comprehensive mechanical properties of the material can be effectively improved.^[^ ^80^ ^]^ In addition to the influence of the alloy‐element type and concentration, temperature is another important factor affecting the stacking fault energy. The stacking fault energy can be reduced in a low‐temperature environment, that is, twin‐induced or phase‐transformation‐induced plasticity can occur at low temperatures.^[^ ^175^, ^176^ ^]^ Recently, a heterogeneous microstructure design has become an effective strategy to improve the strength‐ductility balance. It is characterized by the intentional design of components and regions with wide differences in scale, hardness, or mechanical properties, such as various gradient microstructures,^[^ ^177^, ^178^, ^179^, ^180^ ^]^ bimodal structures,^[^ ^181^, ^182^ ^]^ etc. In the medium‐entropy alloy, the formation of short‐range chemical order can improve the mechanical properties of the alloy.^[^ ^183^, ^184^, ^185^ ^]^ It is conceivable that if the forming elements of nanoprecipitates can be fully precipitated from the HEA matrix, the number of components in the matrix phase will be reduced, which will lead to the formation of the medium‐entropy matrix phase, so that the alloy performance can be improved again through the formation of short‐range chemical order. **Figure** **7** summarizes the design concept of the nanoprecipitate‐strengthened HEA.

The slow diffusion effect in HEAs is still in debate.^[^ ^12^, ^186^, ^187^, ^188^, ^189^, ^190^, ^191^, ^192^, ^193^ ^]^ In the early studies, the original paradigm of slow diffusion in HEAs was verified by the interdiffusion experiment with the Darken manning formalism for the analysis, quasi‐binary approach, and theoretical calculation.^[^ ^186^, ^187^, ^188^ ^]^ It was reported that the interaction between different atoms and the lattice distortion in HEAs seriously affect the effective diffusion rate of atoms.^[^ ^12^ ^]^ Therefore, the slow diffusion effect is regarded as one of the four effects in HEAs.^[^ ^12^ ^]^ Subsequently, the direct experimental evidences were provided through a diffusion couple method by measuring the diffusion coefficients of Co, Cr, Fe, Mn, and Ni in ideal‐solution‐like Co‐Cr‐Fe‐Mn‐Ni alloys, demonstrating the slow diffusion characteristics of HEAs.^[^ ^189^ ^]^ The slow diffusion in HEAs is attributed to the large lattice potential energy fluctuation, which produces more significant atomic traps and blocks, resulting in a higher activation energy of diffusion.^[^ ^189^ ^]^ In addition, the diffusion‐couple experiment of the Al‐Co‐Cr‐Fe‐Ni alloy in the temperature range of 1273–1373 K also confirms the slow diffusion phenomena in HEAs.^[^ ^186^ ^]^ It was stated that the complexity of crystal structures in HEAs has a greater impact on the diffusion coefficient than the chemical composition.^[^ ^186^ ^]^ However, some recent studies have shown that the slow diffusion of elements cannot be observed directly in some HEAs.^[^ ^190^, ^191^, ^192^ ^]^ For example, the measurement of the tracer diffusion coefficient in CoCrFeNi and CoCrFeMnNi HEAs indicated that the diffusion of elements in HEAs are not dependent on the increase in the number of elements, while the type of elements plays a more obvious role.^[^ ^190^ ^]^ It was reported that the interdiffusion coefficients in single‐phase FCC Al6Co19Cr28Fe28Ni19 and Al6Co28Cr19Fe19Ni28 HEAs do not support the correlation between the diffusivity and the configurational entropy of mixing. Also, large potential energy fluctuations may not always result in the sluggish diffusion in HEAs.^[^ ^192^ ^]^ In addition, the kinetic parameters from the MAA (Moleko, Allnatt, and Allnatt) light approach do not support the slow diffusion of all atomic components in HEAs.^[^ ^193^ ^]^ In summary, there is no consensus on the existence of slow diffusion effects in HEAs though it is accepted that the complexity of lattice structures caused by element types plays a more important role in diffusion than the increase in the configuration entropy.^[^ ^186^, ^190^, ^192^ ^]^ On the other hand, the diffusion of elements in alloys can be significantly influenced by the concentration of defects in HEAs, such as vacancies and dislocations. However, it is seldom reported how the complexity of lattice structures and configuration entropy influence the formation of vacancies and dislocations and subsequently their effects on the diffusion rate. It is necessary to further investigate the diffusion effect in HEAs, which will help us have a deep understanding on the nucleation and growth of precipitates in HEAs.

## 5 Prospect

The design strategy of HEAs overturns the traditional perception, which broadens the exploration of new materials. Based on the design concept of HEAs, some new concept materials emerge, such as high‐entropy ceramics.^[^ ^194^, ^195^, ^196^ ^]^

Nanoprecipitates have been proved to be one of the most effective methods to strengthen HEAs. At present, nanoprecipitate‐strengthened HEAs are mainly concentrated in the expanded system of the Cantor alloy, but in theory, it can be applied to different types of HEAs. The first series is the lightweight HEAs. In lightweight HEAs, the high density elements, such as Ni and Co, are not included, but the low‐density Fe, Mn, Cr, Ti, and Al are preferred. A new low‐cost, lightweight Al_1.5_CrFeMnTi HEA was designed by Feng et al.^[^ ^197^, ^198^, ^199^ ^]^ The L2_1_ phase in the light HEA is coherently distributed in the BCC phase. The size, shape, coherency, and spatial distribution of the L2_1_ phase can be changed by annealing. The second series is the refractory HEAs. Refractory HEAs are mainly composed of metal elements with high melting points, which have potential applications in aerospace. Recently, Lei et al.^[^ ^200^ ^]^ optimized the typical TaNbHfZrTi refractory HEA with 2.0 at% O. The tensile yield strength of the (TiZrHfNb)_98_O_2_ HEA reached 1.11 ± 0.03 GPa, and the tensile ductility was 27.66% ± 1.13%. If the nanoprecipitates containing Al and Cr can be introduced into this alloy, its strength may be improved again, and the existing oxidation problem can be alleviated. The third series is the radiation‐resistant HEAs. Compared with other conventional materials, HEAs have no obvious grain‐coarsening phenomenon and have self‐healing ability, especially excellent structural stability, and low radiation‐induced volume swelling under neutron or ion irradiation.^[^ ^201^, ^202^, ^203^ ^]^ Therefore, HEAs are considered as a potential nuclear material for future fission or fusion reactors.^[^ ^201^, ^204^, ^205^ ^]^ When the nanoprecipitates are preset in HEAs, it can be used as a trap to capture the irradiation‐induced vacancies and interstitials, which is expected to improve the radiation resistance. Of course, it is also possible to introduce nanoprecipitates into other types of HEAs to improve the comprehensive mechanical properties.

Moreover, many alloy‐design strategies in the traditional alloys can be introduced to the design of HEAs.^[^ ^9^, ^10^, ^86^, ^206^ ^]^ For example, nanoscale precipitates can be used as a sustainable dislocation source and improve the strength and plasticity of the alloys.^[^ ^86^ ^]^ It is also a promising strategy to improve both the strength and ductility in HEAs utilizing high‐density dislocations. This strategy is originated from the design of deformed and partitioned steels. The high density of dislocations improve the yield strength through dislocation‐forest hardening and the plasticity through the sliding of the existing movable dislocations and the release of the TRIP effect.^[^ ^9^ ^]^ There is also another strategy to strengthen and ductilize alloys through modulating transformation from FCC to BCC structure. The precipitate characteristics (size and spacing) can be applied to control spatial confinement and, in turn, conventional strengthening, martensitic phase transformation, and transformation‐induced plasticity without changing the alloy composition. The composition of the alloy matrix can be modified to tune the chemical driving force for martensite formation to change the spatial confinement effects on strength and phase transformation.^[^ ^206^ ^]^ On the other hand, the unique chemical‐boundary engineering method can be introduced in the design of HEAs. The very sharp chemical discontinuities in the continuous lattice region can limit the strong barrier of the subsequent phase transformation in the ultra‐fine (submicron) domain, achieving high strength and good ductility (>20%).^[^ ^10^ ^]^

At present, the development of HEAs is still in its infancy, and some basic problems that challenge conventional alloy theories, models, and methods have yet to be resolved, such as the slow diffusion effect, the interactions between intrinsic heterogeneities and precipitates in HEAs, etc. In terms of alloy strengthening, various methods are also attempted in HEAs, in which nanoscale‐precipitation strengthening is proved to be one of the most effective strategies. Although nanoprecipitates can effectively strengthen HEAs, many critical issues are still exposed during the alloy design and the corresponding processing. For instance, almost all the nanoprecipitates designed to strengthen effectively HEAs are derived from superalloys, and no unique nanoprecipitates have been found. Furthermore, some negative effects, including the nanoprecipitate coarsening or crystal‐structure transformation during high‐temperature service, can degrade the performance. It is also a critical issue that the matrix phase designed for certain required functional properties cannot meet the expected comprehensive mechanical properties at the same time.

## Conflict of Interest

The authors declare no conflict of interest.
